# Surgical treatment and outcome of intracranial hemangiosarcoma in two dogs: case series

**DOI:** 10.3389/fvets.2026.1778366

**Published:** 2026-04-10

**Authors:** Nina Biundo, Dominic J. Marino, Patrick Roynard

**Affiliations:** 1 College of Veterinary Medicine, The Ohio State University, Columbus, OH, United States; 2 Long Island Veterinary Specialists, Plainview, NY, United States

**Keywords:** brain surgery, canine, case series, doxorubicin, hemangiosarcoma, intracranial, outcome

## Abstract

The treatment and outcome of central nervous system hemangiosarcoma has rarely been documented in dogs, seldom intracranially. We present the first two canine cases of ante-mortem diagnosis, surgical & post-operative adjunctive treatment, and outcome of intracranial hemangiosarcoma presenting as a solitary mass. Each patient originally presented following onset of seizure activity. Both patients underwent magnetic resonance imaging (MRI) of the brain which revealed an intracranial mass. The masses were surgically removed, and histopathological evaluation was consistent with hemangiosarcoma in each case. Both patients underwent screening for primary and metastatic neoplasia, but there was no evidence of neoplasia elsewhere in the body, raising a suspicion of primary intracranial hemangiosarcoma (not confirmed due to absence of necropsy). One patient solely underwent surgical resection with no additional adjuvant therapy and was humanely euthanized 87 days post-operatively due to worsening of quality of life. The other patient received post-operative adjuvant therapy with doxorubicin. Recurrent cluster seizure activity prompted repeat MRI 280 days post-operatively, which confirmed regrowth. Radiation therapy with stereotactic radiosurgery (SRS, CyberKnife^®^) was pursued on day 310; however, the dog was humanely euthanized due to worsening behavioral changes 314 days post-operatively. This series discusses that surgical resection of this solitary mass is doable and may be associated with good quality of life in the short to intermediate term.

## Introduction

Hemangiosarcoma is an aggressive, malignant canine neoplasm arising from vascular-lining endothelial cells (1), most frequently in the spleen, liver, subcutaneous tissues, and the right atrium and auricle of the heart (2). Very few reports have also described hemangiosarcoma arising primarily from intracranial structures in dogs (3–6). While these reports showed that hemangiosarcoma should not be overlooked as a differential diagnosis for a solitary intracranial mass, there are currently no accounts of treatment modalities and their associated outcomes. Among canine neoplasia, hemangiosarcoma has one of the poorest prognoses due to its aggressive biological behavior, high likelihood of metastasis, and insidious nature often resulting in the masking of clinical signs until late in the disease (7). The recommended course of treatment varies based on the tissue of origin, the severity of local invasion, and the degree of metastasis. Wide surgical excision is generally the treatment of choice for localized, non-visceral hemangiosarcoma due to its lower metastatic rate (8), whereas adjunctive chemotherapy is recommended for visceral hemangiosarcoma due to its more aggressive nature and higher likelihood of metastasis (9).

To our knowledge, this is the first detailed report of ante-mortem diagnosis, surgical management, and outcome of two canine cases of intracranial hemangiosarcoma presenting as a single mass.

## Case reports

Each patient’s neurologic condition was localized to the forebrain due to seizure activity. Medical records from all veterinary visits following their initial presentation for seizures and until euthanasia were recorded and reviewed retrospectively. The remaining physical and neurologic examination findings for each recorded visit are included in Supplementary Table 1. Additional diagnostics that were performed throughout the continuation of care are included in Supplementary Table 2. The individualized treatments that each patient received are discussed in each case description below and expanded upon in Supplementary Table 3.

### Case A

A 6-year-old, male-neutered, Bernese Mountain Dog, initially presented to the referring veterinarian for acute onset of hyporexia. The patient was noted to have Horner’s syndrome of the left eye, suspected to be idiopathic in origin (10), but was otherwise apparently healthy on physical examination. A complete blood count (CBC) and serum biochemistry were performed. The results were relatively unremarkable, but abnormalities included minimal hemoglobinemia (20.4 g/dL; reference range [12.1–20.3 g/dL]), hypernatremia (158 mEq/L [139–154 mEq/L]), and hyperchloremia (122 mEq/L [102–120 mEq/L]). Total T4 was unremarkable. A Heartworm-Antigen-Anaplasma-*Borrelia Burgdorferi*-Ehrilichia Antibody Test Kit was also negative. Thoracic radiographs were normal with no evidence of pulmonary pathology nor metastasis.

**Figure 1:**
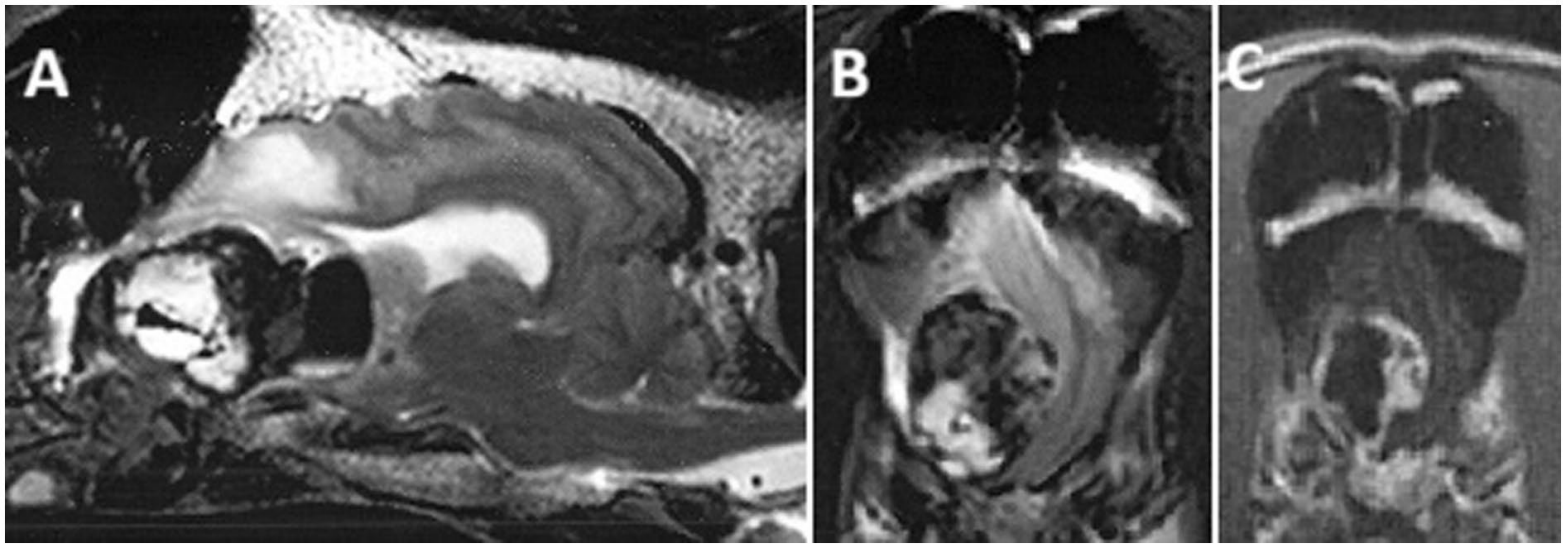
MR images of a 6-year-old MN Bernese mountain dog (patient A) with primary brain hemangiosarcoma: **(A)** Sagittal T2W, **(B)** transverse T2W, and **(C)** transverse T1W post-gadolinium at the olfactory bulbs/rostral frontal lobe. Note the large heterogeneous mass in the frontal lobe with areas of T2W hypointensity and strong peripheral ring enhancement.

Three days later, the patient presented for emergency evaluation due to lethargy, bilateral pelvic limb weakness, intermittent epistaxis, dyspnea, and generalized seizures with inappropriate urination, hypersalivation, and decreased responsiveness during these events. Physical examination abnormalities at the time of emergency presentation included anisocoria with miosis and ptosis of the left eye, moderate ceruminous debris and erythema in both ears, and bilateral pelvic limb stiffness and weakness with mild effusion and thickening palpated on both stifles. The only abnormalities on neurologic examination reported at this time were postural reaction deficits of the left limbs. A CBC was repeated at the time of emergency presentation which revealed a relative lymphopenia (11.8%[12–30%]), relative monocytosis (8.0% [2–4%]), relative erythrocytosis (RBC: 9.1×10^12^/L [5.5–8.5×10^12^/L]; HCT: 59.46% [37–55%]), and hemoglobinemia (20.7 g/dL [12–18 g/dL]). Evaluations of PTT, prothrombin time, platelet counts, fibrinogen, and d-dimers were all within normal limits. Abdominal ultrasound did not reveal any evidence of neoplasia or other intra-abdominal pathologies. An MRI of the brain performed with a 3.0 T system^1^ revealed a large, solitary, intra-axial, contrast-enhancing, cavitary mass with severe peritumoral edema originating from the right olfactory and right frontal lobe, compressing the ventricles. The mass was heterogenous with overall T2W hyperintensity, T1W hypointensity and strong peripheral contrast-enhancement after gadolinium, but also large central area of T2W hypointensity and mixed T1W intensity devoid of contrast enhancement, suggestive of a hemorrhagic neoplasm, common MRI features with previous report of hemangiosarcoma, albeit solitary (6) (Figure 1). Differential diagnoses prioritized neoplasia such as a glioma (possibly high-grade due to contrast enhancement), brain metastasis, and rare variants such as hemangiosarcoma, embryonal tumors or intra-axial histiocytic sarcoma. The dog was hospitalized with intravenous fluid therapy (LRS + 20 mEq KCl/L at 2.16 mL/kg/h IV), prednisone (0.43 mg/kg PO every 12 h), diazepam (0.11 mg/kg IV as needed), and seizure watch. The patient did well with no seizure activity and was discharged the following day on tapering dose of prednisone (0.43 mg/kg every 12 h for 7 days, then 0.43 mg/kg every 24 h for 7 days) while awaiting surgical removal of the intracranial mass.

Within 1 week of initial diagnosis via MRI, surgical excision of the 3 × 3 cm intracranial mass was performed via a free-handed, pneumatic burr-assisted, classical transfrontal craniectomy, as described classically (11–13). The outer table of the frontal bone was not replaced (12), but the periosteum was elevated from midline bilaterally during the approach and sutured over the defect during closure, preventing cosmetic deformation. No breach of the cribriform plate was seen perioperatively with 3.5x magnification microsurgical loupes, and no neoplastic tissue identified in the frontal sinus/nasal cavity. After durotomy, the mass was intra-axial, of soft and heterogenous consistency, with large hemorrhagic areas and poorly defined boundaries. The patient recovered uneventfully from anesthesia and was hospitalized for supportive care, pain management, and monitoring following the procedure. Post-operatively and throughout hospitalization, the patient received intravenous fluid therapy (Normosol + 20 mEq KCl/L + 20 mg/L metoclopramide at 2.81 mL/kg/h IV), cefazolin (21.6 mg/kg IV every 8 h), buprenorphine (0.008–0.01 mg/kg IV as needed up to every 6 h), tramadol (2.16 mg/kg PO every 8 h), levetiracetam (38.9 mg/kg IV once, then 43.2 mg/kg PO every 8 h), zonisamide (4.86 mg/kg PO every 12 h), prednisone (0.54 mg/kg PO every 12 h), famotidine (0.54 mg/kg IV every 12 h), and diazepam (0.25 mg/kg IV once). He did not have any seizure activity during hospitalization and was discharged 5 days post-operatively with cephalexin (21.6 mg/kg PO every 8 h for 10 days), famotidine (0.43 mg/kg PO every 24 h for 7 days), prednisone (0.43 mg/kg PO every 24 h for 7 days, then every 48 h for 7 days), tramadol (2.16 mg/kg PO every 8 h for 7 days), levetiracetam (43.2 mg/kg PO every 8 h for 30 days), and zonisamide (4.86 mg/kg PO every 12 h for 30 days). Post-operative MRI was offered to assess completeness of resection but was declined due to finances.

The initial histopathology report (see Supplementary material) described this olfactory bulb/frontal lobe mass as a poorly delineated neoplasm, composed of fusiform to stellate mesenchymal cells with coarsely stippled chromatin and a moderate to high mitotic rate, forming vascular spaces and capillaries filled with blood and fibrin thrombi, with multifocal hemorrhage and necrosis. The microscopic diagnosis from this sample was consistent with hemangiosarcoma of the olfactory brain region, suggested to either have originated within the calvarium or metastasized to the location. A second histopathology performed by an academic laboratory (Cornell University) was consistent with a diagnosis of hemangiosarcoma, describing neoplastic mesenchymal cells exhibiting marked anisocytosis and anisokaryosis, forming irregular vascular channels within the neuroparenchyma (see Supplementary material). Thoracic radiographs, abdominal ultrasound, and other aforementioned diagnostics were unremarkable or not clinically significant (see Supplementary Table 2), resulting in consideration for primary intracranial hemangiosarcoma, local extension of an extracranial origin or metastatsis from a primary unimaged anatomical location.

Chemotherapy was recommended as an adjunctive treatment based on histological results but was declined. First recheck examination occurred 14 days post-operatively, and the patient was reportedly doing very well with no seizure activity. The first post-operative seizure occurred 17 days post-operatively. He presented in focal status epilepticus 56 days post-operatively following inadequate administration of anti-epileptics at home. During his subsequent hospitalization, the patient experienced four focal facial seizures and two generalized seizures. Ensuring proper anti-epileptic dosages after this event resulted in adequate seizure management for this patient. Changes to the patient’s medical protocol were made based on frequency and severity of seizure activity along with the development of other clinical signs, with a total of 8 post-operative visits for various reasons (rechecks, seizure-related emergency, euthanasia), detailed in Supplementary Table 3. The patient was ultimately euthanized 87 days post-operatively due to decreased appetite, uncontrollable epistaxis, and a concern for the patient’s quality of life.

### Case B

A 9-year-old, female-spayed, Siberian Husky, presented for a newly acquired seizure disorder that started 5 months prior to referral with a total of 3 isolated generalized seizures and one cluster event. Physical examination findings included over conditioning with a body condition score of 8/9, stiffness in all four limbs, and bilateral pelvic limb lameness. No deficits were reported on neurologic examination, but pain was elicited on ventral palpation of the caudal cervical vertebrae, presumably secondary to the later noted intracranial neoplasia (14–17).

**Figure 2:**
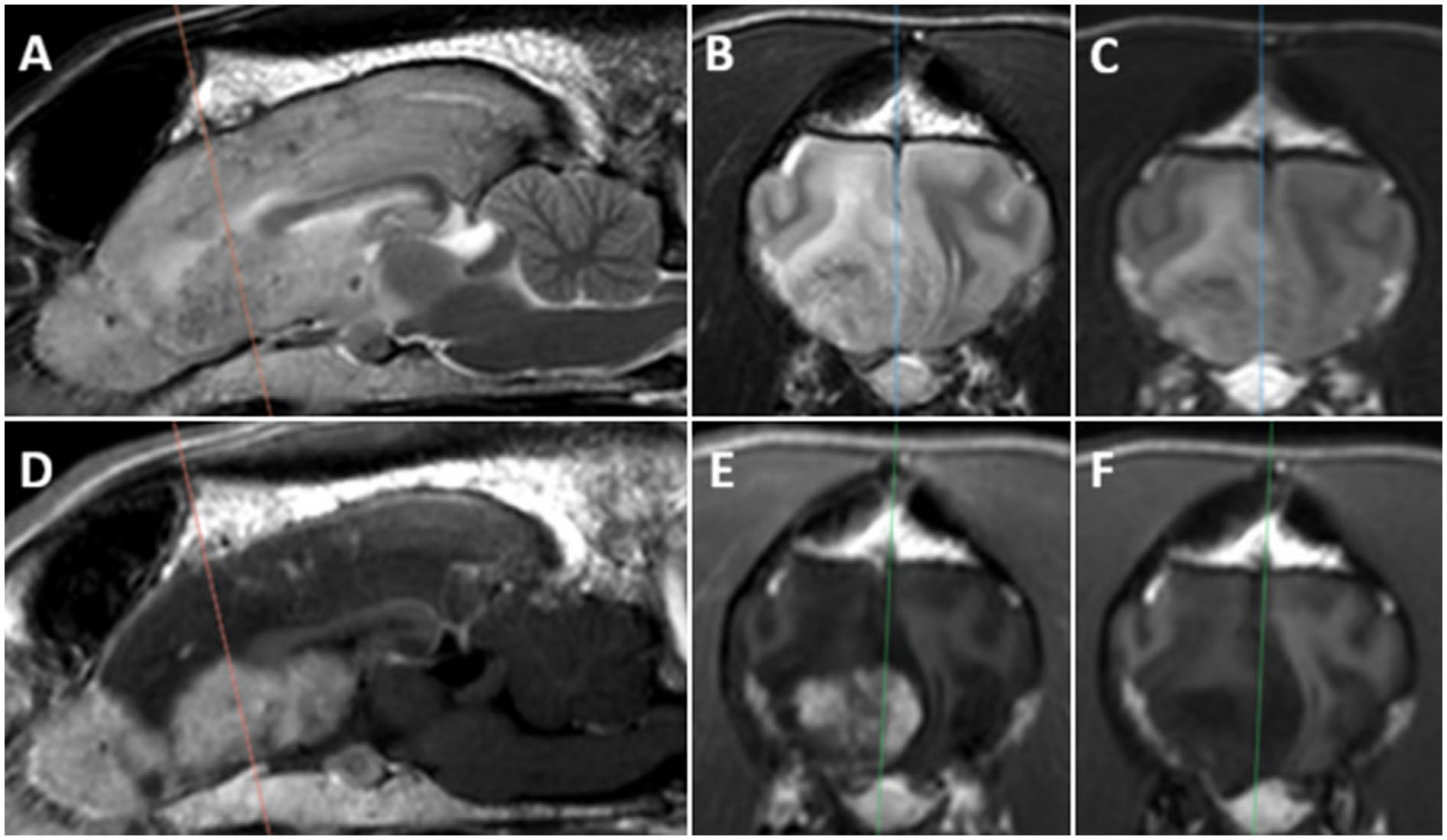
MR images of a 9-year-old FS Siberian Husky (patient B) with primary brain hemangiosarcoma: **(A)** Sagittal T2W, **(B)** transverse T2W, **(C)** transverse FLAIR, **(D)** sagittal T1W post-gadolinium, **(E)** transverse T1W post-gadolinium, **(F)** transverse T1W pre-gadolinium. The *orange line* on sagittal images indicates the corresponding transverse sections; the *blue* and *green lines* indicate corresponding sagittal sections for T2W and T1W images, respectively. Note the large heterogeneous mass in the ventral part of the right frontal lobe and right olfactory bulb, with multiple small foci of T2W hypointensity and heterogenous marked contrast enhancement on post-gadolinium images.

A CBC revealed a minimal lymphopenia (1.03 K/uL [1.05–5.1 K/uL]). Serum biochemistry revealed hypernatremia (168 mmol/L [144–160 mmol/L]), hyperchloremia (126 mmol/L [109–122 mmol/L]), increased alanine aminotransferase (139 U/L [10–125 U/L]), increased alkaline phosphatase (425 U/L[23–212 U/L]), and hypercholesterolemia (431 mg/dL [110–320 mg/dL]). Prior diagnostics are listed in Supplementary Table 2. Thoracic radiographs were unremarkable with no evidence of primary pathology nor metastatic lesions. MRI of the brain performed with similar 3.0 T system (see text footnote 1) revealed a large, T2 heterogeneous (hyperintense with smaller foci of hypointensity than case A), T1 hypointense, markedly contrast enhancing mass in the right olfactory bulb and right frontal lobe, similar in appearance to that reported in case A (Figure 2). On images, the mass equivocally extended through the cribriform plate and into the caudal recess of the right nasal cavity, with also a mass effect resulting in a right-to-left falx shift in the olfactory bulbs and frontal lobes. Additionally, a second, small, contrast-enhancing mass was noted in the pituitary fossa. Differentials for the olfactory bulb mass prior to histopathology sampling focused on neoplasms, including carcinoma, meningioma, histiocytic sarcoma, and olfactory neuroblastoma, among others.

The Siberian Husky was initially prescribed zonisamide (5.65 mg/kg PO every 12 h) to manage her seizure events. The dosage of zonisamide was increased to 11.30 mg/kg PO every 12 h during pre-operative visits due to increased frequency of episodes. Prednisone (0.42 mg/kg PO every 12 h for 2 weeks) was added to her therapy prior to surgical intervention to reduce vasogenic edema. A free-handed, pneumatic burr-assisted classical transfrontal craniectomy was performed 27 days after the initial MRI, with similar technique as case A. Despite concern of the mass equivocally extending to the cribriform plate on MR images, this was not appreciated at the time of surgery with, as for case A, an intact cribriform plate and no neoplastic tissue identified in the frontal sinus/nasal cavity with 3.5x magnification microsurgical loupes. After durotomy, a Cavitron Ultrasonic Surgical Aspirator (CUSA) was utilized to target and remove the diseased tissue (see Discussion). As for case A, the mass was intra-axial, of soft and heterogeneous consistency, with hemorrhagic areas and poorly defined boundaries. The patient did well during the procedure, recovered uneventfully from general anesthesia, and was hospitalized for a few days following surgery for ongoing monitoring and care. Post-operative management included IV fluids, analgesics, anti-inflammatory medication (prednisone) and anti-seizure medications (Zonisamide, Levetiracetam), however precise dosages and exact duration of hospitalization could not be retrieved accurately from retrospective review of available medical records. A post-operative MRI was offered to evaluate completeness of resection but was declined.

Histopathology described an unencapsulated, invasive neoplasm composed of fusiform mesenchymal cells that formed variably sized vascular channels filled with blood and fibrin. The cells were supported by a thin, fibrous stroma, had hyperchromatic fusiform nuclei with coarsely stippled chromatin, and the nucleoli were small, but conspicuous. The final report was consistent with hemangiosarcoma (see Supplementary material). Given this diagnosis, echocardiogram and abdominal ultrasound were pursued to search for a primary neoplasm and were unremarkable (see Supplementary Table 2). Due to the lack of supporting evidence demonstrating neoplasia elsewhere in the body, this mass was presumed to be of intracranial origin.

**Figure 3:**
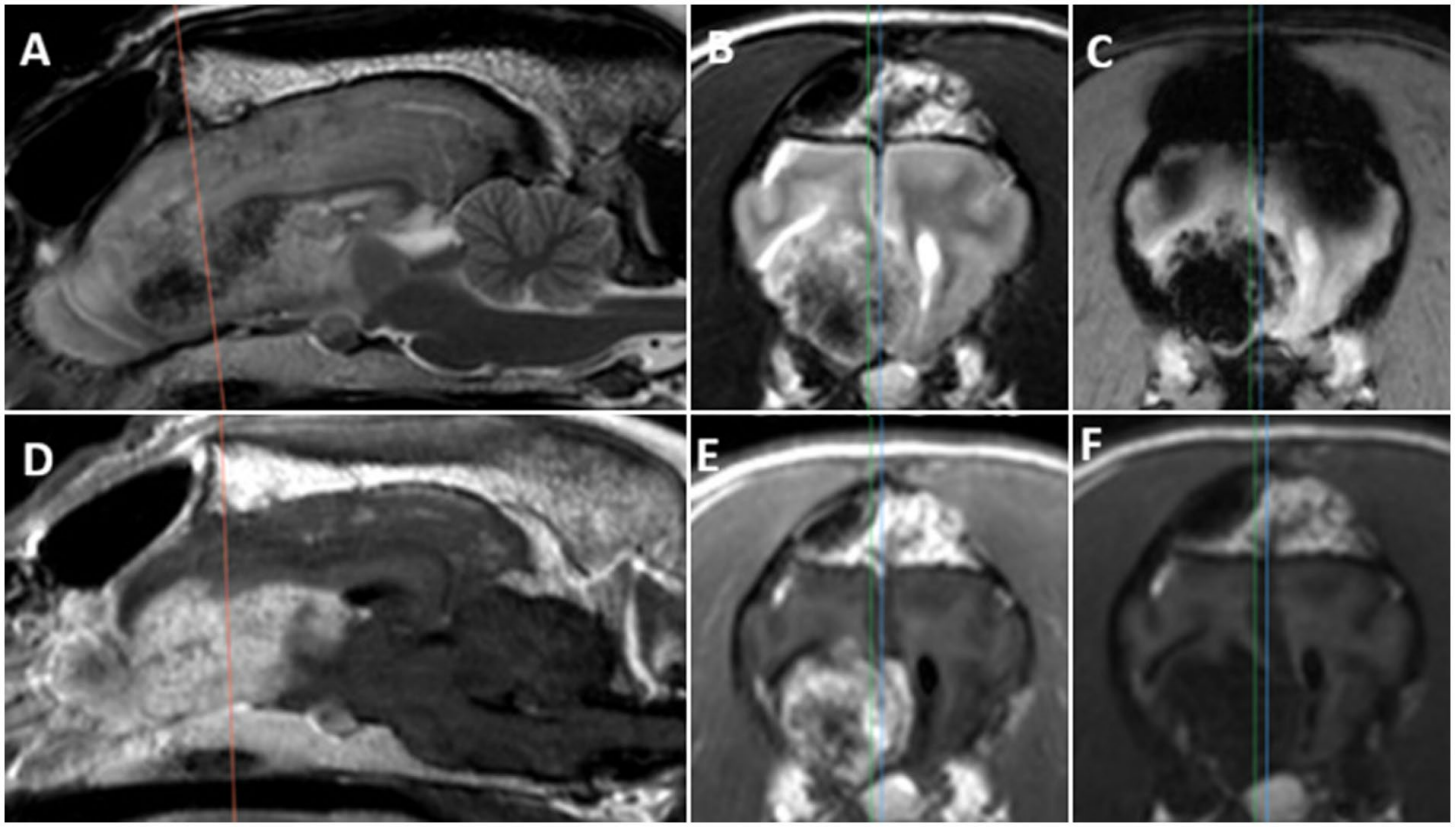
MR images of a 9-year-old FS Siberian Husky (patient B) with regrowth of primary brain hemangiosarcoma 10 months post-surgery: **(A)** Sagittal T2W, **(B)** transverse T2W, **(C)** transverse FFE, **(D)** sagittal T1W post-gadolinium, **(E)** transverse T1W post-gadolinium, **(F)** transverse T1W pre-gadolinium. The *orange line* on sagittal images indicates the corresponding transverse sections; the *blue* and *green lines* indicate corresponding sagittal sections for T2W and T1W images, respectively. Note the large heterogeneous mass in the ventral part of the right frontal lobe and right olfactory bulb, with wider foci of T2W hypointensity associated with signal void on FFE, and heterogenous marked contrast enhancement on post-gadolinium images.

Recheck examination 8 days post-operatively showed good recovery with resolution of seizure activity, abnormal mentation, and pacing behavior. Adjunctive therapy included chemotherapy, which began 20 days post-operatively and utilized doxorubicin administered IV over 5 sessions at 3 weeks interval at a dosage of 30 mg/m^2^, as reported previously (18, 19). The patient experienced cluster seizures and status epilepticus 55 days post-operatively after reportedly missing one dose of levetiracetam. She then had one generalized seizure 157 days post-operatively and a cluster event of two focal facial seizures and one generalized seizure which prompted hospitalization 229 days post-operatively. Her next episode of seizure activity occurred 264 days post-operatively with relapse of abnormal mentation and hyperesthesia persistent post-ictally. Repeat MRI of the brain 280 days post-operatively confirmed regrowth of the mass. The mass appeared very similar to its initial appearance, with heterogeneous aspect on T2 images, including multiple larger areas of T2 hypointensity associated with signal void on FFE (Figure 3). This, in conjunction with the significant contrast enhancement post-gadolinium, was very suggestive of this mass being highly vascular and a recurrence of the primary intracranial hemangiosarcoma as it is a described MRI feature (6). However, histopathology was not obtained. Abdominal ultrasound and thoracic radiographs performed in close association with this MRI did not show evidence of neoplasia/metastasis. Throughout the entire follow-up, the patient received different anti-epileptics, steroids, non-steroidal anti-inflammatories, and other medications, based on frequency/severity of seizures and other clinical signs. The timeline and explanation of these alterations in medical therapy are further detailed in Supplementary Table 3. Stereotactic radiosurgery with CyberKnife^®^ technology was elected 310 days post-operatively, but the patient underwent only 1 treatment and was humanely euthanized 314 days post-operatively due to worsening behavioral changes and quality of life.

## Discussion

Despite imaging and histologic confirmation aligning with standard diagnostic criteria and that of prior study (6, 20), this case series is subject to limitations based on its retrospective nature and small sample size. The former can result in a risk of information bias (e.g., recall bias), with missing/incomplete records and data (e.g., the patients were not systematically examined by a veterinary neurologist, histological pictures and immunohistochemistry unavailable retrospectively).

In case A, no cause for the transient epistaxis at presentation for seizure pre-operatively was identified [normal coagulation parameters (Supplementary Table 2) + no tumor identified in the nasal cavity], which aligns with a prior canine study reporting unknown as the most common etiology (67/176 ≈ 38%). Seizure-induced trauma and neoplastic tissue in the nasal cavity (although not visualized) were also considered as they are the two most commonly identified etiologies in, respectively, 29 and 30% of dogs (21). Also in case A, although the signs of Horner’s syndrome (typically not associated with lesions of the rostral forebrain) introduce the possibility of a structural, metastatic lesion along the oculosympathetic pathway, Horner’s has already been reported with intracranial tumors affecting the olfactory area of the brain (without necessarily inferring causality, e.g., nasal adenocarcinoma) (22). Furthermore, the absence of signs from such hypothetical lesion (no cervical myelopathy, no cervical mass, no sign of bulla disease on MRI) and the overwhelming frequency of idiopathic etiology of Horner’s when compared to neoplastic (respectively 123 versus 6 cases in a 2019 review) (10) seem compellingly in favor of an idiopathic origin over metastatic. Although numerous diagnostics (e.g., repeated thoracic radiographs, abdominal ultrasounds and radiographs, echocardiogram; see Supplementary Table 2) and metastasis-free follow-up for case B oppose a metastatic origin, consideration was given to a nasal cavity origin with local invasion. However, in both cases, the intact cribriform plate and inner table of the frontal bone at surgery, the absence of tumoral material in the frontal sinus and nasal cavity, the intraparenchymal aspect of the tumor on images and at surgery, and the lack of olfactory epithelium described on histology reports are all contrary to this hypothesis. Therefore, a presumptive diagnosis of primary intracranial hemangiosarcoma was suspected. However, post-mortem examination was not performed in either case to confirm the suspicion that the intracranial masses were primary in origin, thus a primary extracranial lesion with intracranial invasion or metastasis cannot be entirely excluded.

The goals of treatment in canine hemangiosarcoma include tumor removal, or at least, cytoreduction, and delaying metastasis (7–9). More aggressive treatment approaches for hemangiosarcoma in companion animals include any combination of surgical resection, radiation, chemotherapy, and more recently, immunotherapy (7–9, 23). Nevertheless, these multimodal and more invasive approaches are rarely curative. A recent study demonstrated that immunotherapy with peptide-based vaccination in dogs with aggressive hemangiosarcoma undergoing surgery and chemotherapy improved time to disease progression and overall survival (23). Although this study focused on splenic, cardiac, osseous, and retroperitoneal hemangiosarcoma, this novel therapeutic approach may prove to be beneficial to intracranial hemangiosarcoma in the future.

**Figure 4:**
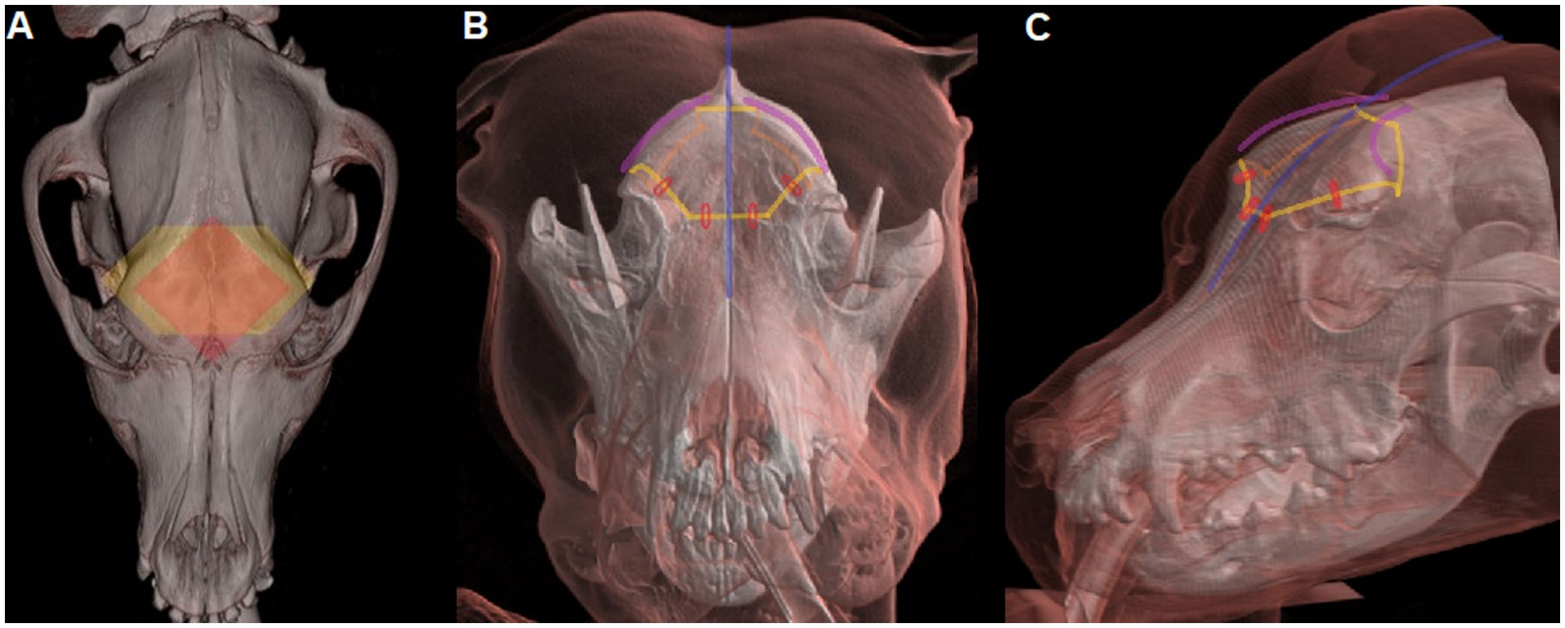
**(A)** Comparison of the bone window in classical transfrontal craniotomy (*red diamond*) compared to bifrontal/modified transfrontal craniotomy (*yellow hexagon*). **(B)** Frontal and **(C)** Oblique view of bifrontal craniotomy: the *blue line* indicates midline cutaneous incision; the *curved purple lines* indicate the bilateral incisions of the temporalis muscles/fascia along the temporal lines prior to craniotomy of the outer layer of the frontal bone, including the temporal lines and lateral limits of the frontal sinus (*yellow lines*). The large bone piece is placed back in anatomical position during closure and secured with suture of the temporalis fascia on each temporal line dorsally (*purple lines*) and non-absorbable sutures ventrally (*red circles*). Reproduced with permission from Roynard P. *Chapter 44: Craniectomies and Brain Surgery*, in Small Animal Surgery, Fossum T (Ed), 6th edition, Elsevier, 2026.

Hemangiosarcoma currently carries a varied prognosis dependent on its tissue of origin, with the most common locations again noted to be the spleen, liver, subcutaneous tissues, and the right atrium and auricle of the heart (2). Prognosis for splenic hemangiosarcoma has been well-documented and varies dependent upon clinical stage and addition of immunotherapy with consensus statements describing a MST of 170 days with surgery and adjuvant chemotherapy (7). Hepatic hemangiosarcoma is not as thoroughly documented, with case reports describing death 7 days after surgical excision versus 201 days after diagnosis with surgical excision and aggressive chemotherapy (24, 25). Median survival time for subcutaneous hemangiosarcoma ranges from 8 months to > 3 years when treated with surgery, doxorubicin-based chemotherapy and/or radiation therapy (26, 27). Cardiac hemangiosarcoma has been documented to have an average survival ranging from 45 days to 5 months with surgical resection alone and 164 days when combined with chemotherapy (7). Hence, despite variations based on the tissue affected and excluding certain subcutaneous forms, prognosis remains guarded even with multimodal therapy. In both our cases, surgical management was considered to obtain a histological diagnosis and due to the solitary aspect of the mass. Prognosis following surgical resection of intracranial neoplasia in veterinary and human patients is influenced by multiple factors. They include tumor-specific parameters (e.g., tumoral type and biological behavior—discussed above), patient-specific parameters (e.g., comorbidities and their treatment) and treatment-specific considerations, further divided into surgical considerations (e.g., completeness of tumor removal) vs. adjuvant therapies (e.g., type of chemotherapy and/or radiotherapy) (13). Both patients underwent a similar procedure, transfrontal craniectomy, performed by one (DM, case A) or two (DM & PR) of the authors, hence not interpreted as a source of difference, with however the use of an ultrasonic aspirator in case B. A surgical aspirator provides preferential fragmentation of high-water content tissue (e.g., neoplasia) coupled with an irrigation-suction system. Its use in intracranial surgery has been advocated due to higher safety for peritumoral tissues and increased completeness of tumor removal (28). This possibly influenced the outcome, as prior reports using CUSA for meningioma removal have documented longer survival time than reported otherwise (29–31). A modified transfrontal/bifrontal approach, mobilizing the entire outer bony margins of the frontal sinus, has been described by several authors in the last 10 years (13, 32–35) and has become the authors’ preference when tackling large masses located in the frontal lobes, due to the wider brain exposure (Figure 4). Retrospectively, this may have afforded more complete tumor removal and would be the authors’ recommendation if tackling similar lesions surgically at this time.

Although definitive conclusions cannot be drawn from only 2 cases but as observed with other forms of hemangiosarcoma (see above), it is worth noticing that the patient treated with adjunctive therapies (five rounds of chemotherapy/doxorubicin and one round of stereotactic radiation) had a post-operative survival time 227 days longer than the patient treated with surgery alone (87 vs. 314 days). Since the patient was euthanized only 4 days after stereotactic radiotherapy, it cannot be the origin of the difference in outcome. The differing survival time among patients suggests, as for other locations of hemangiosarcoma, that multimodal approaches combining surgical resection (including CUSA and possibly other technologies maximizing tumor resection) and adjunctive therapies may improve prognosis in patients with intracranial hemangiosarcoma, but further studies are necessary to support this hypothesis and identify ideal management.

The aim of this series is to document antemortem diagnosis, treatment, and outcome of solitary intracranial hemangiosarcoma in two canine patients. While imaging characteristics and histology reports were used to support this diagnosis, the novelty of this report lies in the conclusion that surgical resection of solitary intracranial hemangiosarcoma is doable and may be associated with good quality of life in the short to intermediate term.

## Data Availability

The original contributions presented in the study are included in the article/Supplementary material, further inquiries can be directed to the corresponding authors.

## Footnotes

1 Achieva, Philips Medical System, Andover, MA 01810, United States.
