# Supplementary material for: Surgical treatment and outcome of intracranial hemangiosarcoma in two dogs: case series
Source: Front Vet Sci. 2026 Apr 10;13:1778366. doi: 10.3389/fvets.2026.1778366 (PMC13106059; doi:10.3389/fvets.2026.1778366)
Supplement: Supplementary file 1 [file Table_1.docx]

**Table 1.** General Timeline (day 0 = intracranial surgery leading to diagnosis of brain hemangiosarcoma) and Clinical Examination Findings at Each Presentation (Summary).

| **Patient** | **Post-Operative Day (POD)** | **Presenting Complaint** | **Physical and Neurologic Examination Findings** |
| --- | --- | --- | --- |
| A | 9 days pre-operatively | Hyporexia, hypersalivation, dyspnea | Horner’s syndrome of the left eye |
| A | 6 days pre-operatively | Lethargy, weakness, epistaxis, intermittent hypersalivation and dyspnea, decreased appetite, inappropriate urination, and generalized seizure activity | Anisocoria, miosis and ptosis of the left eye, moderate ceruminous debris and erythema in both ears, mild joint effusion and thickened stifles bilaterally, postural reaction deficits of the left limbs |
| A | POD 0 | Surgical intake | Not retrievable |
| A | POD 14 | Recheck | Pelvic limb weakness |
| A | POD 17 | Seizure activity: Focal facial seizure that became generalized | Generalized weakness, ataxia, mydriasis and sluggish pupillary light reflex in the right eye  *An additional focal facial and two generalized seizures occurred throughout hospitalization* |
| A | POD 31 | Recheck | Horner’s syndrome of the left eye |
| A | POD 56 | Seizure activity: Focal status epilepticus  *Owner reported previous cluster event of three seizure episodes within four hours* | Facial twitching, ptosis of the left eye, bilaterally absent menace response, ptyalism, post-ictal tetra ataxia  *An additional four focal facial and two generalized seizures occurred throughout hospitalization* |
| A | POD 59 | Hyporexia | Horner’s syndrome of the left eye |
| A | POD 82 | Hyporexia and lethargy  *Owner reported one generalized seizure occurred the day before presentation* | Serosanguinous nasal discharge, bilaterally absent menace response, anisocoria, absent pupillary light reflex of the right eye |
| A | POD 86 | Epistaxis | Epistaxis right nares, anisocoria |
| A | POD 87 | Humane euthanasia elected | Full neurologic examination not performed |
| B | 178 days pre-operatively | Generalized seizure with one-hour post-ictal period | Mild dental calculus, over conditioning, stiffness in all limbs, pelvic limb lameness, pain elicited on palpation of caudal cervical vertebrae |
| B | 36 days pre-operatively | Behavioral changes and hyporexia  *Owner reported six seizures had occurred within six months* | Erythema and ceruminous debris in both ears, mild dental disease, over-conditioning, intermittent bilateral pelvic limb lameness, pain elicited on palpation of caudal cervical vertebrae |
| B | 28 days pre-operatively | Diagnostic workup | Bilateral pelvic limb lameness |
| B | 20 days pre-operatively | Recheck | Left pelvic limb lameness |
| B | POD 0 | Surgery intake | Left pelvic limb lameness |
| B | POD 8 | Recheck  *Owner reported abnormal behaviors (pacing, whining) had resolved* | Blood-tinged nasal discharge |
| B | POD 15 | Recheck | Unremarkable |
| B | POD 20 | Chemotherapy 1/5 | Unremarkable |
| B | POD 29 | Chemotherapy bloodwork | Unremarkable |
| B | POD 41 | Chemotherapy 2/5 | Unremarkable |
| B | POD 55 | Status epilepticus  *Six generalized seizures within one hour without regaining full consciousness between events*  *Owner reported missing a dose of levetiracetam* | Bilaterally absent menace response |
| B | POD 61 | Recheck | Increased upper airway sounds, bilateral pelvic limb lameness, sedate mentation, moderate para-ataxia, paraparesis, postural deficits in all four limbs |
| B | POD 75 | Chemotherapy 3/5 | Focal erythema of muzzle |
| B | POD 96 | Chemotherapy 4/5 | Focal erythema of muzzle |
| B | POD 117 | Chemotherapy 5/5 | Focal erythema of muzzle and lipomatous mass in the cervical inlet |
| B | POD 146 | Recheck | Left pelvic limb lameness and soft, mobile, subcutaneous mass at the level of the cervical inlet, focal erythema of muzzle |
| B | POD 157 | No associated visit | One generalized seizure reported |
| B | POD 166 | Cough | Left pelvic limb lameness and soft, mobile, subcutaneous mass at the level of the cervical inlet, focal erythema of muzzle |
| B | POD 229 | Two focal facial seizures and one generalized seizure  *Intermittent focal facial seizures occurred during evaluation* | Miotic pupils, gas distension of abdomen, generalized weakness, difficulty ambulating, delayed conscious proprioception  *Additional two focal facial seizures occurred during hospitalization* |
| B | POD 264 | Recheck | Left pelvic limb lameness and soft, mobile, subcutaneous mass at the level of the cervical inlet |
| B | POD 274 | Seizure activity: one event with subsequent disorientation and hyperesthesia | Agitated mentation, hyperesthesia, and soft, mobile, subcutaneous mass at the level of the cervical inlet |
| B | POD 280 | Repeat MRI | Agitated mentation, hyperesthesia, and soft, mobile, subcutaneous mass at the level of the cervical inlet |
| B | POD 288 | Recheck  *Owner reported behavioral changes (whining, pacing), one focal seizure, and one generalized seizure since previous visit* | Unremarkable |
| B | POD 296 | Diagnostic workup | Limited examination; unremarkable |
| B | POD 307 | RT planning | Stiff gait, weakness in pelvic limbs |
| B | POD 309 | CyberKnife radiation | Stiff gait, weakness in pelvic limbs |
| B | POD 314 | Humane euthanasia elected | Records not available |
