# Supplementary material for: Surgical treatment and outcome of intracranial hemangiosarcoma in two dogs: case series
Source: Front Vet Sci. 2026 Apr 10;13:1778366. doi: 10.3389/fvets.2026.1778366 (PMC13106059; doi:10.3389/fvets.2026.1778366)
Supplement: Supplementary file 2 [file Table_2.docx]

**Table 2.** Diagnostic Tests and Reported Results Throughout Treatment and Monitoring (day 0 = intracranial surgery leading to diagnosis of brain hemangiosarcoma)

| **Patient** | **Post-Operative Day (POD)** | **Diagnostics Performed** | **Results (abnormal values reported)** |
| --- | --- | --- | --- |
| A | 9 days pre-operatively | Thoracic radiographs | No significant findings |
|  |  | Complete blood count | Hemoglobinemia (20.4 g/dL; reference range 12.1 – 20.3 g/dL) |
|  |  | Serum Biochemistry | Hypernatremia (158 mEq/L; reference range 139 – 154 mEq/L) and hyperchloremia (122 mEq/L; reference range 102 – 120 mEq/L). |
|  |  | SNAP 4Dx | Negative |
| A | 6 days pre-operatively | Complete blood count | Increased red blood cell count (9.1x10^12^/L; reference range 5.5 – 8.5x10^12^/L), increased hematocrit (59.46%; reference range 37 – 55%), increased mean corpuscular hemoglobin concentration (MCHC) (34.8 g/dl; reference range 31 – 34 g/dl), and hemoglobinemia (20.7 g/dl; reference range 12 – 18 g/dl). |
|  |  | Coagulation panel (activated partial thromboplastin time, prothrombin time, platelet count, quantitative fibrinogen, d-dimer) | All values within normal limits |
|  |  | Abdominal ultrasound | No significant findings |
|  |  | MRI | Contrast-enhancing, cavitary mass noted in right olfactory region with peritumoral edema |
| A | POD 0 | Complete blood count | Monocytosis (2.06 K/uL; reference range 0.30 – 2.00 K/uL) |
| A | POD 2 | Histopathology (Antech) | Consistent with hemangiosarcoma |
| A | POD 5 | Histopathology (Cornell) | Consistent with hemangiosarcoma |
| A | POD 59 | Serum biochemistry | Increased alanine aminotransferase (206 U/L; reference range 10 – 100 U/L), increased alkaline phosphatase (273 U/L; reference range 23 – 212 U/L), hypercholesterolemia (361 mg/dL; reference range 110 – 320 mg/dL), hypernatremia (161 mmol/L; reference range 144 – 160 mmol/L), and hyperchloremia (129 mmol/L; reference range 109 – 122 mmol/L) |
| B | 178 days pre-operatively | Complete blood count | All values within normal limits |
|  |  | Serum biochemistry | Hyperglycemia (131 mg/dl; reference range 63 – 124 mg/dl), hypernatremia (151.4 mmol/L; reference range 139 – 151 mmol/L), hyperglobulinemia (4.5 g/dl; reference range 2 – 3.6 g/dl), hypercholesterolemia (>450 mg/dl; reference range 120 – 310 mg/dl), and increased alkaline phosphatase (182 U/l; reference range 0 – 140 U/l) |
|  |  | Thoracic radiographs | No significant findings |
| B | 28 days pre-operatively | Complete blood count | Lymphopenia (1.03 K/uL; reference range 1.05 – 5.1 K/uL) |
|  |  | Serum biochemistry | Hypernatremia (168 mmol/L; reference range 144 – 160 mmol/L), hyperchloremia (126 mmol/L; 109 – 122 mmol/L), increased alanine aminotransferase (139 U/L; reference range 10 – 125 U/L), increase alkaline phosphatase (425 U/L; reference range 23 – 212 U/L), and hypercholesterolemia (431 mg/dL; reference range 110 – 320 mg/dL) |
|  |  | Thoracic radiographs | No significant findings |
|  |  | MRI | Mass noted in right olfactory region and frontal cortex; additional mass detected in pituitary fossa |
| B | POD 0 | Histopathology | Consistent with hemangiosarcoma |
|  |  | Serum biochemistry | Hypernatremia (161 mmol/L; reference range 144 – 160 mmol/L) and hypokalemia (3.4 mmol/L; reference range 3.5 – 5.8 mmol/L) |
| B | POD 8 | Abdominal ultrasound | No significant findings |
|  |  | Echocardiogram | No significant findings |
| B | POD 29 | Complete blood count | Decreased red blood cell count (4.88 M/uL; reference range 5.65 – 8.87 M/uL), mild macrocytic, normochromic regenerative anemia (HCT: 36.4%; reference range 37.3 – 61.7%) (reticulocytes: 212.8 K/uL; reference range 10 – 110 K/uL) (MCV: 74.6 fL; reference range 61.6 – 73.5 fL), hemoglobinemia (12.2 g/dL; reference range 13.1 – 20.5 g/dL), and monocytosis (1.13 K/uL; reference range 0.16 – 1.12 K/uL) |
| B | POD 41 | Complete blood count | Decreased red blood cell count (5.59 M/uL; reference range 5.65 – 8.87 M/uL), reticulocytosis (127.5 K/uL; reference range 10 – 110 K/uL), and a leukocytosis (27 K/uL; reference range 5.05 – 16.76 K/uL) characterized by a neutrophilia (23.64 K/uL; reference range 2.95 – 11.64 K/uL) and basophilia (0.18 K/uL; reference range 0 – 0.1 K/uL) |
| B | POD 55 | PCV/TS | 43%/9.0 g/dL; lipemic serum |
|  |  | Complete blood count | Unremarkable |
|  |  | Serum biochemistry | Hyperglycemia (131 mg/dl; reference range 75 – 125 mg/dl), increased alkaline phosphatase (182 U/l; reference range 0 – 140 U/l), hypercholesterolemia (>450 mg/dl; reference range 120 – 310 mg/dl), and hyperglobulinemia (4.5 g/dl; reference range 2 – 3.6 g/dl) |
|  |  | SNAP 4Dx | Negative |
|  |  | Abdominal radiographs | No significant findings |
|  |  | Thoracic radiographs | Spondylosis deformans. No significant findings |
| B | POD 75 | Complete blood count | Increased mean corpuscular volume (73.6 fL; reference range 61.6 – 73.5 fL) and basophilia (0.21 K/uL; reference range 0 – 0.1 K/uL) |
| B | POD 96 | Complete blood count | Decreased red blood cell count (5.56 M/uL; reference range 5.65 – 8.87 M/uL) and increased mean corpuscular volume (73.9 fL; reference range 61.6 – 73.5 fL) |
| B | POD 117 | Complete blood count | Decreased red blood count (5.59 M/uL; reference range 5.65 – 8.87 M/uL) and neutrophilia (11.86 K/uL; reference range 2.95 – 11.64 K/uL) |
| B | POD 166 | Thoracic radiographs | Spondylosis deformans. No significant findings |
| B | POD 229 | Serum Biochemistry | Hypercholesterolemia (486 mg/dL; reference range 110 – 320 mg/dL), increased alanine aminotransferase (450 U/L; 10 – 125 U/L), increased alkaline phosphatase (1,886 U/L; reference range 23 – 212 U/L) |
| B | POD 231 | Phenobarbital levels | Within mid-therapeutic range (Trough at 21.5 ug/mL; reference range 15 – 45 ug/mL; Peak at 25 ug/mL; reference range 15-45 ug/mL) |
|  |  | Zonisamide trough | Low therapeutic range (19.2 ug/mL; reference range 10 – 40 ug/mL) |
| B | POD 264 | Serum biochemistry | Increased BUN (30 mg/dL; reference range 7 – 27 mg/dL), hyperchloremia (123 mmol/L; reference range 109 – 122 mmol/L), hypercholesterolemia (463 mg/dL; reference range 110 – 320 mg/dL), and increased alkaline phosphatase (1,497 U/L; reference range 23 – 212 U/L) |
|  |  | Thoracic radiographs | Bilateral glenohumeral osteoarthritis, spondylosis deformans, diffuse bronchointerstitial pattern. No evidence of pulmonary nodules or metastasis. |
| B | POD 274 | Phenobarbital trough | Within therapeutic range (21.1 ug/mL; reference range 15 – 45 ug/mL) |
|  |  | Zonisamide trough | Over therapeutic range (44.1 ug/mL; reference range 10 – 40 ug/mL) |
| B | POD 280 | Complete blood count | Decreased red blood cell count (5.14 M/uL; reference range 5.65 – 8.87 M/uL), increased mean corpuscular volume (75.1 fL; reference range 61.6 – 73.5 fL), and eosinopenia (0.02 K/uL; reference range 0.06 – 1.23 K/uL) |
|  |  | Serum biochemistry | Increased BUN (29 mg/dL; reference range 7 – 27 mg/dl), hypernatremia (170 mmol/L; reference range 144 – 160 mmol/L), hyperchloremia (129 mmol/L; reference range 109 – 122 mmol/L), increased alanine aminotransferase (141 U/L; reference range 10 – 125 U/L), increased alkaline phosphatase (>2,000 U/L; reference range 23 – 212 U/L), hypercholesterolemia (469 mg/dL; 110 – 320 mg/dl), and increased lipase (5,029 U/L; reference range 100 – 1,800 U/L) |
|  |  | MRI | Mass detected in right rostral cerebrum |
| B | POD 296 | Abdominal Ultrasound | Hepatomegaly with rounded margins; suspected secondary to phenobarbital administration. Hyperechoic foci on spleen; suspected to be incidental mineralization or myelolipomas. No significant findings indicative of neoplasia. |
| B | POD 307 | CT | Performed for radiation therapy planning purposes; finalized report unavailable |
