# Supplementary material for: Surgical treatment and outcome of intracranial hemangiosarcoma in two dogs: case series
Source: Front Vet Sci. 2026 Apr 10;13:1778366. doi: 10.3389/fvets.2026.1778366 (PMC13106059; doi:10.3389/fvets.2026.1778366)
Supplement: Supplementary file 3 [file Table_3.docx]

**Table 3.** Additions and Alterations to Medical Protocol Throughout Treatment with Interpretations of Clinicians’ Clinical Reasoning (Summary) (day 0 = intracranial surgery leading to diagnosis of brain hemangiosarcoma)

| **Patient** | **Post-Operative Day** | **Medications administered and/or prescribed** | **Clinical Reasoning** |
| --- | --- | --- | --- |
| A | 9 days pre-operatively | Cephalexin 21.60 mg/kg PO every 12 hours | Empirical treatment for suspected otitis externa |
| A | 6 days pre-operatively | Prednisone 0.43 mg/kg PO every 12 hours for 7 days, then decreased to every 24 hours for 7 days, diazepam 0.11 mg/kg intravenously as needed, and LRS + 20 mEq Kcl/L at 2.16 ml/kg/hr IV | Anti-inflammatory steroid, anxiolytic therapy, and maintenance fluids during hospitalization |
| A | POD 0 | Normosol + 20 meq kcl/L + 20 mg/L metoclopramide at 2.81 ml/kg/hr IV, buprenorphine 0.008 – 0.01 mg/kg IV as needed up to every 4 hours, cefazolin 21.60 mg/kg IV every 8 hours, tramadol 2.16 mg/kg PO every 8 hours, levetiracetam 38.88 mg/kg IV once, then 43.20 mg/kg PO every 8 hours, zonisamide 4.86 mg/kg PO every 12 hours, prednisone 0.54 mg/kg PO every 12 hours, famotidine 0.54 mg/kg PO every 12 hours, and diazepam 0.25 mg/kg IV given once while in hospital  Discharged with cephalexin 21.60 mg/kg PO every 8 hours for 10 days, famotidine 0.43 mg/kg PO every 24 hours for 7 days, prednisone 0.43 mg/kg PO every 24 hours for 7 days, then decrease to every 48 hours for 7 days, tramadol 2.16 mg/kg PO every 8 hours for 7 days, then decrease to every 12 hours for 7 days, levetiracetam 43.2 mg/kg PO every 8 hours for 7 days, and zonisamide 4.86 mg/kg PO every 12 hours for 30 days | Post-operative protocol including maintenance fluids, pain management, an anti-inflammatory steroid, a prophylactic antibiotic, anti-epileptics, and an H2-receptor antagonist to decrease potential reflux, nausea, and vomiting following general anesthesia |
| A | POD 14 | Deracoxib 1.75 mg/kg PO every 24 hours | Alleviate pelvic limb weakness |
| A | POD 17 | Diazepam 0.47 mg/kg IV as needed, tramadol 2.34 mg/kg PO every 12 hours, zonisamide 4.68 mg/kg PO every 12 hours, and levetiracetam 23.41 mg/kg PO every 8 hours for 30 days | Immediate seizure control in hospital and long-term anti-epileptic protocol |
| A | POD 56 | Zonisamide 5.84 mg/kg PO every 12 hours for 30 days, levetiracetam 24.32 mg/kg PO every 8 hours for 30 days, and diazepam 1.07 mg/kg as needed | Anti-epileptic protocol increased due to seizure activity |
| A | POD 59 | Maropitant 1.56 mg/kg PO every 24 hours for 5 days and Denamarin | Antiemetic to combat nausea and decreased appetite; hepatic supplement added following discovery of increased liver enzymes |
| A | POD 82 | Famotidine 0.40 mg/kg PO as needed up to every 12 hours | Plan to alleviate nausea and inappetence |
| A | POD 86 | Intranasal epinephrine | Vasoconstrictor to combat epistaxis |
| B | 96 days pre-operatively | Zonisamide 5.65 mg/kg PO every 12 hours | Initiated anti-epileptic therapy after reported cluster event of three seizures within 24 hours |
| B | 65 days pre-operatively | Zonisamide 8.47 mg/kg PO every 12 hours | Increased anti-epileptic therapy after additional seizure event |
| B | 39 days pre-operatively | Zonisamide 11.30 mg/kg PO every 12 hours | Increased anti-epileptic therapy after additional seizure event |
| B | 20 days pre-operatively | Prednisone 0.42 mg/kg PO every 12 hours for 2 weeks | Anti-inflammatory steroid while awaiting surgery |
| B | POD 0 | Levetiracetam 21.12 mg/kg PO every 8 hours, tramadol 2.11 mg/kg PO every 8 hours, and famotidine 0.56 mg/kg PO every 12 hours | Post-operative protocol including pain management, an additional anti-epileptic, and an H2-receptor antagonist to decrease potential reflux, nausea, and vomiting following general anesthesia |
| B | POD 15 | Prednisone 0.42 mg/kg every 24 hours for 2 weeks, then every 48 hours for 2 weeks | Tapering steroid |
| B | POD 20 | Doxorubicin 30 mg/m2, maropitant 2.25 mg/kg PO as needed, and metronidazole 14.08 mg/kg PO every 12 hours for 5 days | Chemotherapy initiation with additional medications to manage side effects of therapy such as nausea, vomiting, and diarrhea |
| B | POD 41 | Doxorubicin 30 mg/m2, maropitant 2.15 mg/kg PO as needed, and metronidazole 13.47 mg/kg PO every 12 hours for 5 days | Chemotherapy with additional medications to manage side effects of therapy such as nausea vomiting, and diarrhea |
| B | POD 55 | Midazolam 0.51 mg/kg IV and levetiracetam 28.73 mg/kg IV given in hospital  Increased levetiracetam 32.33 mg/kg PO every 8 hours, added phenobarbital 2.79 mg/kg PO every 12 hours, and instructed to continue administering zonisamide as previously prescribed at 11.49 mg/kg PO every 12 hours | Immediate seizure control in hospital and alterations to long-term anti-epileptic protocol |
| B | POD 75 | Doxorubicin 30 mg/m2, maropitant 2.13 mg/kg PO as needed, and metronidazole 13.33 mg/kg PO every 12 hours for 5 days | Chemotherapy with additional medications to manage side effects of therapy such as nausea vomiting, and diarrhea |
| B | POD 96 | Doxorubicin 30 mg/m2, maropitant 2.17 mg/kg PO as needed, and metronidazole 13.59 mg/kg PO every 12 hours for 5 days | Chemotherapy with additional medications to manage side effects of therapy such as nausea vomiting, and diarrhea |
| B | POD 117 | Doxorubicin 30 mg/m2, maropitant 2.35 mg/kg PO as needed, carprofen 2.11 mg/kg PO every 24 hours, and metronidazole 14.66 mg/kg PO every 12 hours for 5 days | Chemotherapy with additional medications to manage reported arthritis and side effects of therapy such as nausea vomiting, and diarrhea |
| B | POD 229 | Diazepam 0.40 mg/kg IV and levetiracetam 40.00 mg/kg IV given in hospital  Discharged with zonisamide 10.67 mg/kg PO every 12 hours, phenobarbital 2.59 mg/kg PO every 12 hours, and levetiracetam 30 mg/kg PO every 8 hours | Immediate seizure control in hospital and alterations to long-term anti-epileptic protocol |
| B | POD 231 | Zonisamide 16.95 mg/kg PO every 12 hours | Increased dose following test results showing zonisamide levels were in low-therapeutic range |
| B | POD 274 | Carprofen discontinued to begin prednisone 0.56 mg/kg PO every 12 hours for 7 days, then every 24 hours thereafter | Concern for tumor reoccurrence |
| B | POD 280 | Mannitol given intravenously at unknown dose in hospital  Discharged with prednisone 0.56 mg/kg PO every 24 hours, gabapentin 8.50 mg/kg PO every 8 to 12 hours, and trazodone 2.82 mg/kg PO every 12 hours | Osmotic diuretic administered following identification of cerebral edema on MRI, tapering steroid, and additional medications provided to address inflammation, pain, and anxiety |
| B | POD 288 | Prednisone 0.56 mg/kg PO every 12 hours | Steroid increased to combat inflammation and associated pain |
| B | POD 309 | Cyberknife radiation | Stereotactic radiation precisely targeted at the reoccurring mass |
