# Supplementary material for: Surgical treatment and outcome of intracranial hemangiosarcoma in two dogs: case series
Source: Front Vet Sci. 2026 Apr 10;13:1778366. doi: 10.3389/fvets.2026.1778366 (PMC13106059; doi:10.3389/fvets.2026.1778366)
Supplement: Supplementary file 4 [file Data_Sheet_1.pdf]

CASE A - HISTOLOGY 1

ANTECH DIAGNOSTICS

800-872-1001

Long Island Vet Specialist PC
ANTECH Acct No. [redacted]

[redacted]

Doctor MARINO

Owner	Pet Name	Species	Breed	Sex	Pet Age	Chart#
[redacted]	[redacted]	Canine	Bouvier des Flandres	CM	7Y	[redacted]

Test Requested	Results	Reference Range	Units

HISTOPATHOLOGY, FULL WRITTEN REPORT

Biopsy

Microscopic Description: Sections of a poorly delineated neoplastic mass from the olfactory region of the brain are examined. Neural tissue is not observed in the sample. It is composed of fusiform to stellate mesenchymal cells forming vascular spaces and capillaries filled with blood and fibrin thrombi. The cells have hyperchromatic nuclei with coarsely stippled chromatin and a moderate to high mitotic rate. The cytoplasm blends imperceptibly into the surrounding fine collagenous matrix. There is multifocal hemorrhage and necrosis within the neoplasm.

Microscopic Findings: Hemangiosarcoma, olfactory region.

Comment: Hemangiosarcomas are locally invasive neoplasm with a high incidence of recurrence and distant metastasis. The neoplasm may have originated in the calvarium or metastasized to this location. The prognosis is guarded to poor.

[redacted]DVM, PhD, DACVP [redacted]@antechmail.com
(800) 872-1001 ext. 3950 (516) 326-3950

REPORT NOTES:

RT SIDE OF BRAIN TUMOR

[redacted] 10:25AM ō aware that prelim report cancerous tumor. Final report coming from Cornell probably in about a week. ōh w/ oncology consult once final report available. [redacted]

[illegible]

[illegible]

Cornell University
Animal Health Diagnostic Center
240 Farrier Road, Cornell University, Ithaca, NY 14853
Ph: 607-253-3900 Fax: 607-253-3943
http://diagcenter.vet.cornell.edu

Finalized Report

Owner: [redacted]

Accession Number: [redacted]

CASE A - HISTOLOGY 2

Long Island Veterinary Specialists, P C - (11526)
Dr Dominic Marino
163 S Service Rd
Plainview, NY 11803
(516) 501-1700

[redacted]

Reference Number: [redacted]

ANATOMIC PATHOLOGY

Department of Biomedical Sciences
Phone: 607-253-3319 | Fax: 607-253-3357

History

Description: Chronic lethargic, Hc. weakness. Horner's Syndrome - MRI - right side brain mass. Olfactory region.

Tissue Submitted: Brain tumor, right side olfatory region.

1 [redacted] - Canine Bemese Mountain Dog Castrate

Histopathology

Histologic Diagnosis
Brain: Hemangiosarcoma

Final Comments
The neoplasm is composed of neoplastic mesynchymal cells forming irregular vascular channels, consistent with a diagnosis of hemangiosarcoma. Additional imaging is recommended in this patient to rule out that the lesion is metastatic from a primary neoplasm located elsewhere most commonly the right auricle, spleen or liver. Both submitted samples show similar histologic features; however, neuroparenchyma is only seen in the Cassi II collected sample and shows excellent histological morphology.

Description
Mass, Nos: Brain, regular sample, 6 sections (slides 1&2): Examined are 6 sections of brain mass from the right side of the olfactory region sampled in a regular fashion, per submission. The sections are composed of moderately cellular, moderately demarcated nodules of neoplastic mesynchymal cells that form irregular spaces and channels amidst a fibrovascular. The neoplastic cells have extensive pale eosinophilic cytoplasm and large round to oval nuclei with coarsely stippled to vesicular chromatin and a prominent nucleolus. The neoplastic cells exhibit marked anisocytosis and anisokaryosis; 9 often large and bizarre mitotic figures are noted in ten high magnification fields. Small numbers of hemosiderin-laden macrophages are present amongst neoplastic cells with large aggregates of hemorrhage, fibrin and necrosis. Minimal appreciable neuroparenchyma is present in the examined sections.

Brain, Cassi II sample, 3 sections (slide 3): Examined are 3 sections of a Cassi II sample of a mass from the right side of the olfactory region, per submission. One section contains a nodular, moderately cellular mesynchymal neoplasm composed of neoplastic cells similar to those described in slides 1&2. Fewer vascular channels are present within the nodule, and regular multinucleate cells are noted. One section is composed of histologically normal neuroparenchyma with mild perivascular hemorrhage.

Pathologist [redacted] DVM, Ph.D, DACVP

Resident Pathologist [redacted] DVM, Ph. D

CASE B - HISTOLOGY

ANTECH DIAGNOSTICS

800-872-1001

Long Island Veterinary Specialist PC
ANTECH Acct No. [redacted]

[redacted]

Doctor MARINO

Owner	Pet Name	Species	Breed	Sex	Pet Age	Chart#
[redacted]	[redacted]	Canine	Siberian Husky	SF	10Y	[redacted]

Test Requested	Results	Reference Range	Units

HISTOPATHOLOGY, FULL WRITTEN REPORT

History:

Brain tumor. History of seizures.
MRI findings: Region 1: brain--there is a large, complexly intense, fairly uniformly contrast enhancing soft tissue mas in the right olfactory and frontal cortex. There is a large mass effect and a significant right-to-left falx shift caused by this mass. The mass extends through the right side of the cribriform plate and into the caudal recess of the right nasal cavity.
The right optic nerve appears swollen and it is infiltrated and/or compressed just rostral to its junction at the optic chiasm.
There is a second, small, contrast enhancing mass in the pituitary fossa.
No other abnormalities are seen.
Clinical assessment: rule-out neoplasm, i.e. paranasal meningioma, falx meningioma, other-right olfactory and frontal cortex. Rule out 2nd mass, i.e. pituitary macroadenoma, other. Rest: WNL.
-
Received: 1 x 1 cm tissue.

Biopsy

DESCRIPTION/MICROSCOPIC FINDINGS/COMMENTS:

Microscopic Description:
Sections of a mass from the frontal cortex are examined. There is an unencapsulated, invasive neoplasm composed of fusiform mesenchymal cells that form variably sized vascular channels. Many are filled with blood and fibrin. The cells have hyperchromatic fusiform nuclei with coarsely stippled chromatin. Nucleoli are small, but conspicuous. There is a low mitotic rate (0-1/hpf). The cells are supported by a thin fibrous stroma. Hemorrhage is observed in the intervening stroma.

Microscopic Findings:
Hemangiosarcoma, frontal cortex.

Comment:
The mass within the frontal cortex is a hemangiosarcoma. Since the patient has no history of hemangiosarcoma elsewhere, this appears to be a primary hemangiosarcoma of the brain. Primary hemangiosarcoma of the brain are rather uncommon, most are sites of metastasis [1]. The prognosis is poor.

Reference:
[1] Waters DJ, et al. Intracranial lesions in dogs with hemangiosarcoma. J Vet Intern Med. 1989 Oct-Dec;3(4):222-30.

PATHOLOGIST:

[redacted] DVM, PhD, DACVP
[redacted]@antechmail.com
(800) 872-1001 ext. 3950
